# Whole-genome fetal and maternal DNA methylation analysis using MeDIP-NGS for the identification of differentially methylated regions

**DOI:** 10.1017/S0016672316000136

**Published:** 2016-11-11

**Authors:** ANNA KERAVNOU, MARIOS IOANNIDES, KYRIAKOS TSANGARAS, CHARALAMBOS LOIZIDES, MICHAEL D. HADJIDANIEL, ELISAVET A. PAPAGEORGIOU, SKEVI KYRIAKOU, PAVLOS ANTONIOU, PETROS MINA, ACHILLEAS ACHILLEOS, MARIA NEOFYTOU, ELENA KYPRI, CAROLINA SISMANI, GEORGE KOUMBARIS, PHILIPPOS C. PATSALIS

**Affiliations:** 1 Translational Genetics Team, The Cyprus Institute of Neurology and Genetics, Nicosia, Cyprus; 2 NIPD Genetics, Nicosia, Cyprus; 3 Department of Cytogenetics and Genomics, The Cyprus Institute of Neurology and Genetics, Nicosia, Cyprus

## Abstract

DNA methylation is an epigenetic marker that has been shown to vary significantly across different tissues. Taking advantage of the methylation differences between placenta-derived cell-free DNA and maternal blood, several groups employed different approaches for the discovery of fetal-specific biomarkers. The aim of this study was to analyse whole-genome fetal and maternal methylomes in order to identify and confirm the presence of differentially methylated regions (DMRs). We have initially utilized methylated DNA immunoprecipitation (MeDIP) and next-generation sequencing (NGS) to identify genome-wide DMRs between chorionic villus sampling (CVS) and female non-pregnant plasma (PL) and peripheral blood (WBF) samples. Next, using specific criteria, 331 fetal-specific DMRs were selected and confirmed in eight CVS, eight WBF and eight PL samples by combining MeDIP and in-solution targeted enrichment followed by NGS. Results showed higher enrichment in CVS samples as compared to both WBF and PL samples, confirming the distinct methylation levels between fetal and maternal DNA for the selected DMRs. We have successfully implemented a novel approach for the discovery and confirmation of a significant number of fetal-specific DMRs by combining for the first time MeDIP and in-solution targeted enrichment followed by NGS. The implementation of this double-enrichment approach is highly efficient and enables the detailed analysis of multiple DMRs by targeted NGS. Also, this is, to our knowledge, the first reported application of MeDIP on plasma samples, which leverages the implementation of our enrichment methodology in the detection of fetal abnormalities in maternal plasma.

## 1. Introduction

Chromosomal aneuploidies are the most common causes of genetic defects during the first trimester, which constitutes the major reason for pregnant women considering prenatal diagnosis (Hassold *et al.*, 2007). Prenatal screening tests are usually performed in the first or second trimester of pregnancy by combining ultrasound findings with biochemical screening, providing a risk factor with limited sensitivity and specificity. Currently, the gold standard in prenatal diagnosis is provided by invasive procedures, such as chorionic villus sampling (CVS) or amniocentesis, which are associated with a significant risk of fetal loss (Hulten *et al.*, 2003). Thus, in the last decade, great interest has been shown towards the development of noninvasive prenatal testing (NIPT) methodologies that can be offered to all pregnant women without any risk of miscarriage.

The discovery of cellfree fetal DNA (cffDNA) in maternal plasma was pivotal in the development of NIPT (Lo *et al.*, 1997). Recent studies have shown that fetal DNA concentrations in the maternal circulation are estimated to be approximately 10% during the first trimester (Lun *et al.*, 2008). Several research groups utilized the presence of cffDNA in maternal blood and were able to develop different approaches for the identification and utilization of fetalspecific biomarkers for NIPT. Early studies focused on the identification of fetalspecific markers, such as DYS14, RhesusD and paternally inherited polymorphic loci, which are easily distinguishable in maternal circulation (Lo *et al.*, 1998; Daniels *et al.*, 2006). Despite efforts aimed at the discovery of fetalspecific markers that can be detected in all pregnancies irrespective of fetal gender and polymorphisms, the limited amount of cffDNA in the presence of a high maternal background presents a major challenge for the detection of fetal aneuploidies.

DNA methylation is an epigenetic marker that involves the addition of a methyl group on carbon 5 of the cytosine present in CpG dinucleotides. It has been shown that methylation patterns across different tissues vary significantly. As such, tissuespecific differentially methylated regions (DMRs) have been utilized as biomarkers for disease monitoring and prognosis, especially in the field of cancer research (Paulsen & FergusonSmith, 2001; Baylin, 2005; Chan *et al.*, 2013). In the prenatal setting, taking advantage of the methylation differences between the placentaderived cffDNA and the maternal peripheral blood DNA, several groups employed bisulphite conversion and methylationsensitive restriction digestion for the identification of fetalspecific DMRs that can potentially be used for the detection of fetal aneuploidies. These studies resulted in the discovery of only a small number of DMRs, including *SERPINB5, AIRE, SIM2, ERG, U-PDE9A* and *RASSF1A* (Old *et al.*, 2007; Chim *et al.*, 2008).

Methylated DNA immunoprecipitation (MeDIP) in combination with highresolution tiling oligonucleotide array analysis was first introduced in the field of NIPT in 2009 by Papageorgiou *et al.* for the discovery of fetalspecific DMRs. Specifically, more than 2000 DMRs were identified between placental and female nonpregnant peripheral blood (WBF) on each of chromosomes 21, 18, 13, X and Y (Papageorgiou *et al.*, 2009). MeDIP in combination with realtime quantitative polymerase chain reaction (MeDIPqPCR) was later introduced for the quantification of selected DMRs on chromosome 21, resulting in 100% sensitivity and specificity for the detection of trisomy 21 (Papageorgiou *et al.*, 2011). Using the same approach, a second validation study of 175 cases yielded 100% sensitivity and 99·2% specificity for the NIPT of trisomy 21 (Tsaliki *et al.*, 2012). An expansion of the fetal biomarker panel was also introduced by our group, providing the first step towards the development of NIPT assays for trisomy 18 (Ioannides *et al.*, 2014).

Recently, the advent of nextgeneration sequencing (NGS) has revolutionized the development of NIPT (Fan *et al.*, 2012; Kitzman *et al.*, 2012), providing new opportunities for the detection of fetal aneuploidies (Chiu *et al.*, 2008; Lo *et al.*, 2010) and other genetic abnormalities (Chen *et al.*, 2013). In addition, methylated DNA enrichment methods and bisulphite conversion followed by NGS have been utilized to investigate the fetal methylome and its potential use in the development of methylationbased NIPT (Papageorgiou *et al.*, 2009; Lun *et al.*, 2013; Papageorgiou *et al.*, 2014).

In the present study, we have utilized MeDIP in combination with NGS for genomewide fetalspecific DMR identification in CVS, wholeblood nonpregnant female samples (WBF) and female nonpregnant plasma (PL) samples. Using a novel doubleenrichment approach (MeDIP in combination with insolution hybridization enrichment followed by NGS), we have confirmed the presence of a set of 331 DMRs in multiple CVS, WBF and PL samples. The results of this study demonstrate that there is a clear distinction between the methylation levels of fetal and maternal DNA for the selected DMRs. The utilization of a novel doubleenrichment approach in this study provides a significant expansion in the number of fetalspecific biomarkers. This increase in fetal biomarkers sets the foreground for the implementation of our approach in the detection of the most common fetal aneuploidies.

## 2. Materials and methods

### 2.1. Sample collection and DNA extraction

In total, 11 WBF, 10 PL and 11 firsttrimester CVS (11–14 weeks of gestation) were used in this study (Table 1). The study has been approved by the Cyprus National Bioethics Committee and informed written consent was obtained from all participants. All WBF and CVS samples were collected from collaborating centres of the Translational Genetics Team and the Department of Cytogenetics and Genomics at the Cyprus Institute of Neurology and Genetics (Nicosia, Cyprus). PL samples were obtained from Sera Laboratories International Ltd (Sussex, UK).

**Table 1.** Number of samples used in our study

Experimental step	CVS samples	PL samples	WBF samples	Sequencing type
DMR discovery	3	2	3	Wholegenome sequencing
DMR confirmation	8	8	8	331 target capture probes
Total	11	10	11	

CVS: chorionic villus sampling; PL: female nonpregnant plasma sample; WBF: female nonpregnant whole blood.

Peripheral blood was collected from women donors into two 8-mL EDTAcontaining tubes. An average of 8 mL of plasma was isolated using a doublecentrifugation protocol as previously described (Huang *et al.*, 2008).

WBF and CVS samples were used to extract DNA using the QIAamp Blood Midi Kit (Qiagen, Hilden, Germany) and the QIAamp DNA Mini kit (Qiagen), respectively, according to the manufacturer's instructions. DNA from PL samples was extracted using the QIAsymphony DSP Virus/Pathogen Mini Kit (Qiagen).

### 2.2. Experimental design

DMR identification was initially performed on three WBF, three CVS and two PL samples using wholegenome MeDIPNGS. Based on specific criteria (see Section 3.2) we selected 331 DMRs that were found to be hypermethylated in the fetal tissues and hypomethylated in maternal whole blood and plasma. Confirmation of the methylation status of the 331 DMRs was performed on eight CVS, eight WBF and eight PL samples using MeDIP followed by targeted insolution enrichment and NGS.

### 2.3. MeDIP and sequencing library construction

Extracted DNA was quantified by realtime qPCR using the DYS14 and *β*globin loci, as described previously (Zimmermann *et al.*, 2005). Genomic DNA ranging from 18 ng–3 μg was sheared to an average size of 230 bp using the Bioruptor Twin Sonicator (UCD400, Diagenode, Liege, Belgium). For DMR discovery, bluntending and sequencingadaptor ligation were performed prior to MeDIP using NEB Blunting and Ligase enzymes (NEB, Ipswich, UK), as previously described (Meyer & Kircher, 2010; Tsangaras *et al.*, 2014; Koumbaris *et al.*, 2016). For DMR confirmation, bluntending and adaptor ligation were performed using the iDEAL Library Preparation kit (Diagenode), following the manufacturer's protocol.

For DMR discovery, MeDIP was performed on three CVS and three WBF samples using the MagMeDIP kit (Diagenode), according to the manufacturer's protocol. The remaining MeDIP experiments (10 PL, 8 CVS and 8 WBF) were performed using mouse anti5′methylcytosine monoclonal antibody (Eurogentec, Saraing, Belgium), as described previously using 18–30 ng of genomic DNA (Borgel *et al.*, 2012). In summary, hypermethylated DNA was captured for 2 hours at 4 °C using 3 μL of Dynabeads M-280 sheep antimouse IgG magnetic beads (Life Technologies, Carlsbad, CA, USA) and was washed three times with 1700 μl of 1× immunoprecipitation buffer. The captured DNA was subsequently released using 1·4 μl of proteinase K (Roche, Mannheim, Germany) by heating at 50 °C for 30 minutes. Three technical replicates were performed for each sample, which were then pooled prior to the cleanup step. All cleanup reactions were performed using the QIAquick PCR Purification Kit (Qiagen), following the manufacturer's instructions.

After MeDIP, sequencing libraries used for DMR discovery were amplified for 15 cycles as described previously (Meyer & Kircher, 2010; Koumbaris *et al.*, 2016). After MeDIP, these libraries, which were used for the confirmation of selected DMRs, were amplified using the iDEAL Library Preparation Kit (Diagenode), following the manufacturer's protocol.

### 2.4. Design and preparation of target capture probes

OligoAnalyzer 3·1 software was used to design specific primers in order to generate 144–160 bp long target capture probes for each of the selected DMRs (Owczarzy *et al.*, 2008). PrimerBLAST (NCBI) and *in silico* PCR (UCSC Genome browser) were used in order to confirm primer specificity. PCR reactions were performed using MyTaq HS DNA Polymerase (BioLine, London, UK), as described elsewhere (Koumbaris *et al.*, 2016). Capture probes were confirmed by agarose gel electrophoresis and were purified using the QIAquick PCR Purification Kit. The concentration of each probe was measured using the NanoDrop spectrophotometer (Thermo Scientific, Wilmington, MA USA) and probes were pooled equimolarly. Pooled probes were biotinylated and immobilized on streptavidincoated magnetic beads (Thermo Scientific, Vilnius, Lithuania), as previously described (Tsangaras *et al.*, 2014).

### 2.5. Targeted enrichment and sequencing

For targeted enrichment, 700–1200 ng of each barcoded library was mixed with 2× hybridization buffer (Agilent, Santa Clara, CA, USA), 10× blocking agent (Agilent), blocking oligonucleotides (Maricic *et al.*, 2010), human Cot1 (Invitrogen, Carlsbad, CA, USA) and salmon sperm DNA (Invitrogen) (Maricic *et al.*, 2010; Koumbaris *et al.*, 2016). Immunoprecipitated libraries were incubated with the biotinylated capture probes for 48 hours at 66 °C and were eluted by heating, as previously described (Tsangaras *et al.*, 2014). Enriched samples were amplified using outwardbound adaptor primers (Tsangaras *et al.*, 2014) and were quantified using the KAPA Library Quantification KitIllumina (KAPA Biosystems, Boston, MA, USA). The enriched barcoded libraries were then pooled equimolarly and were subjected to pairedend sequencing on an Illumina HiSeq 2500 system (Illumina, San Diego, CA, USA).

### 2.6. Read trimming and sequence alignment

Sequencing reads were trimmed with cutadapt v.1·2 (Martin, 2011), normalized and aligned to the human reference genome GRCh37/hg19 using the BWA v.0.7.4 MEM algorithm (Li & Durbin, 2009). The Picard tool was used to remove duplicate reads and to convert aligned reads to a binary (BAM) file. Only the uniquely aligned and highquality (quality score >30) reads were used in the sequencing analysis. Local realignment and base recalibration was performed with GATK (McKenna *et al.*, 2010). The SAMtools software was used to retrieve the read depth of each base (Li *et al.*, 2009).

### 2.7. Data analysis

#### 2.7.1. DMR discovery

Candidate fetalspecific DMRs were selected according to the following criteria: (a) the regions selected exhibited consistent DNA hypermethylation profiles in all CVS and hypomethylation in all the female nonpregnant tissues; (b) selected regions had preferentially more than two CpG dinucleotides in the DNA sequence; (c) potential DMRs that were in copy number variable regions or in repetitive element regions were excluded; (d) selected DMRs should be located 200 bp away from repetitive elements; and (e) the adjusted (Bonferroni correction) *P* value of the window bins that covered the region and were obtained from the MEDIPS test for methylation differences was less than 0·10.

Pairwise genomewide methylation comparisons between CVS, WBF and PL were performed using the MEDIPS package (Chavez *et al.*, 2010) with window sizes (bins) specified at 100 bp. Window bins with adjusted *P* value <0·1 were considered for subsequent analysis. These bins were merged into consecutive regions (DMRs) after specific criteria selection and filtering.

#### 2.7.2. DMR confirmation

Prior to analyses, all sequenced reads were normalized by employing ‘vertical normalization’. This normalization method equalizes the cumulative read depth of the selected DMRs across all samples.

A mixedeffects analysis of variance model was applied in order to compare the overall methylation levels of the three tissue types (CVS, WBF and PL). The response variable in this model is the read depth, which in our experiments acts as a proxy for the methylation level of each region, while the categorical variable is the sample type and consists of three levels (CVS, WBF and PL). The additional random effect allows for different methylation variability between the different DMRs. Subsequent *posthoc* pairwise comparisons were applied in order to identify the exact nature of the differences between the three tissues. Three pairwise Welch's t tests were applied and the resulting *P* values were corrected using the Holm–Bonferroni method (Holm, 1979).

## 3. Results

### 3.1. DMR discovery using wholegenome DNA methylation analysis of WBF, CVS and PL samples

Three WBF, three CVS and two PL samples were subjected to wholegenome MeDIPNGS analysis to enable genomewide identification of fetalspecific DMRs. The resulting alignment files were used as input for the R package MEDIPS, where the differential coverage between two groups of samples (i.e. CVS vs. WBF and CVS vs. PL) was calculated. Using MEDIPS criteria (see Section 2.7.1), 3574 DMRs were identified in the CVS vs. WBF comparisons, of which 1888 regions showed hypermethylation in CVS and hypomethylation in WBF samples, while 1686 regions showed hypomethylation in CVS and hypermethylation in WBF samples (Table 2). Similarly, 8091 DMRs were identified in the CVS vs. PL comparison, of which 6313 were hypermethylated in CVS and hypomethylated in PL samples, whereas 1778 regions were hypomethylated in CVS and hypermethylated in PL samples (Table 3). Identified DMRs were distributed across all autosomes. Regions on X- and Y-chromosomes were excluded from the analysis.

**Table 2.** DMRs identified between CVS and WBF DNA samples

	No. of DMRs			% CVS versus WBF		
CHR	No. of HYPER in CVS	No. of HYPO in CVS	Total no.	% HYPER in CVS	% HYPO in CVS	% whole genome
chr1	171	175	346	9·06	10·38	9·68
chr2	173	138	311	9·16	8·19	8·70
chr3	95	72	167	5·03	4·27	4·67
chr4	78	51	129	4·13	3·02	3·61
chr5	122	104	226	6·46	6·17	6·32
chr6	139	139	278	7·36	8·24	7·78
chr7	85	81	166	4·50	4·80	4·64
chr8	108	72	180	5·72	4·27	5·04
chr9	37	32	69	1·96	1·90	1·93
chr10	114	122	236	6·04	7·24	6·60
chr11	79	92	171	4·18	5·46	4·78
chr12	112	69	181	5·93	4·09	5·06
chr13	62	76	138	3·28	4·51	3·86
chr14	97	60	157	5·14	3·56	4·39
chr15	46	59	105	2·44	3·50	2·94
chr16	62	50	112	3·28	2·97	3·13
chr17	93	71	164	4·93	4·21	4·59
chr18	37	45	82	1·96	2·67	2·29
chr19	39	60	99	2·07	3·56	2·77
chr20	72	58	130	3·81	3·44	3·64
chr21	34	31	65	1·80	1·84	1·82
chr22	33	29	62	1·75	1·72	1·73
Total	1888	1686	3574			

CHR: chromosome; CVS: chorionic villus sampling; DMR: differentially methylated region; HYPER: hypermethylated; HYPO: hypomethylated; WBF: female nonpregnant whole blood.

**Table 3.** DMRs identified between CVS and PL DNA samples

	No. of DMRs			% CVS versus PL		
CHR	No. of HYPER in CVS	No. of HYPO in CVS	Total no.	% HYPER in CVS	% HYPO in CVS	% whole genome
chr1	587	177	764	9·30	9·96	9·44
chr2	528	163	691	8·36	9·17	8·54
chr3	310	99	409	4·91	5·57	5·05
chr4	200	65	265	3·17	3·66	3·28
chr5	286	116	402	4·53	6·52	4·97
chr6	412	153	565	6·53	8·61	6·98
chr7	311	66	377	4·93	3·71	4·66
chr8	238	90	328	3·77	5·06	4·05
chr9	146	34	180	2·31	1·91	2·22
chr10	368	95	463	5·83	5·34	5·72
chr11	204	123	327	3·23	6·92	4·04
chr12	366	105	471	5·80	5·91	5·82
chr13	168	79	247	2·66	4·44	3·05
chr14	300	80	380	4·75	4·50	4·70
chr15	282	84	366	4·47	4·72	4·52
chr16	237	34	271	3·75	1·91	3·35
chr17	378	33	411	5·99	1·86	5·08
chr18	132	70	202	2·09	3·94	2·50
chr19	215	21	236	3·41	1·18	2·92
chr20	295	55	350	4·67	3·09	4·33
chr21	160	26	186	2·53	1·46	2·30
chr22	190	10	200	3·01	0·56	2·47
Total	6313	1778	8091			

CHR: chromosome; CVS: chorionic villus sampling; DMR: differentially methylated region; HYPER: hypermethylated; HYPO: hypomethylated; PL: female nonpregnant plasma sample.

#### 3.1.1. Methylation differences between fetal and maternal tissue

**Fig. 1.**
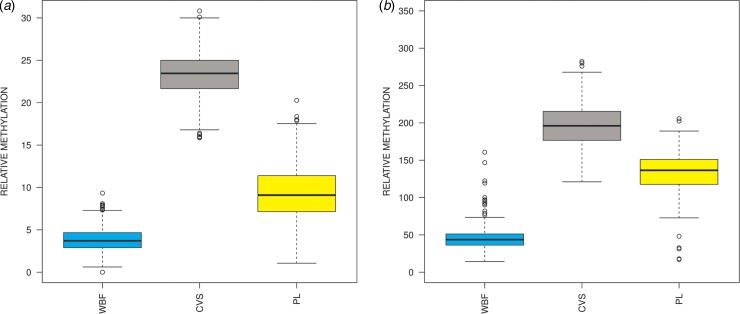
DMR methylation enrichment in CVS, WBF and PL samples. Overall, DMR methylation enrichment in CVS, WBF and PL samples using (*a*) the 1453 common fetalspecific DMRs following MeDIPNGS analysis and (*b*) a subset of 331 fetalspecific DMRs using MeDIP in combination with insolution targeted enrichment followed by NGS. *Posthoc* comparisons showed significant statistical differences between the three tissues (*P* < 2 × 10^−16^) with higher enrichment in CVS compared to maternal DNA, confirming the hypermethylation status of the fetal DNA (CVS) as compared to the maternal DNA (WBF and PL). CVS: chorionic villus sampling; PL: female nonpregnant plasma sample; WBF: female nonpregnant whole blood.

The overlap of the DMRs obtained from the two comparisons (WBF vs. CVS and PL vs. CVS) provided 1453 common fetalspecific DMRs that showed hypermethylation in CVS and hypomethylation in maternal samples. Those ranged from 100 to 2300 bp in length. Comparison of the overall methylation status of the aforementioned DMRs showed a clear distinction of the methylation status between CVS and maternal tissues (WBF and PL), with adjusted *P* < 2 × 10^−16^ for all three pairwise *posthoc* tests (Fig. 1(*a*)). Overall, the DNA methylation enrichment in CVS was greater compared to WBF and PL, while the DNA methylation differences were less pronounced but still statistically significant between CVS and PL than between CVS and WBF.

### 3.2. DMR selection

To further ascertain and characterize the identified biomarkers, a subset of 331 potential fetalspecific DMRs (median length: 149 bp; IQR: 11 bp) was selected based on specific criteria (see Section 2.7.1). In addition, selection was focused on autosomal chromosomes and on regions located in significant regulatory regions such as potential promoters, CpG islands (CGIs) and exonic (coding) regions. Specifically, 294, 64 and 73 DMRs were located within coding regions (67·7% in gene bodies and 32·3% in exons), potential promoters and CGIs, respectively (Table 4 & Table S1).

**Table 4.** Location characteristics of 331 selected DMRs

Chromosome	No. DMRs selected	Gene bodies	Exons	Potential promoters	CGIs
chr1	34	13	6	9	12
chr2	10	10	7	1	3
chr3	10	7	5	1	4
chr4	12	7	5	2	4
chr5	12	8	4	1	5
chr6	13	7	6	3	3
chr7	28	16	5	7	5
chr8	10	9	6	1	4
chr9	9	8	4	1	0
chr10	15	12	5	2	0
chr11	10	8	4	3	4
chr12	12	10	6	2	2
chr13	28	15	3	10	7
chr14	11	4	1	3	2
chr15	9	7	4	1	1
chr16	7	6	2	0	2
chr17	10	9	2	1	0
chr18	31	13	7	3	8
chr19	6	3	1	2	0
chr20	9	5	3	2	3
chr21	39	18	8	9	4
chr22	6	4	1	0	0
Total	331	199	95	64	73

CGI: CpG island; DMR: differentially methylated region.

### 3.3. DMR confirmation

The methylation status of the 331 DMRs was ascertained in a cohort of eight normal CVS, eight WBF and eight PL samples, using MeDIP in combination with insolution targeted enrichment followed by NGS. Overall, methylation comparisons demonstrated significant methylation differences between the three sample types for the 331 selected DMRs (Fig. 1(*b*)). Results showed higher enrichment in CVS samples compared to both WBF and PL samples. Furthermore, a more pronounced methylation difference was apparent between CVS and WBF than between CVS and PL.

The methylation status of the selected DMRs was also compared with previous studies that utilized methylation differences between fetal and maternal tissue for the identification of fetalspecific biomarkers. Common DMRs were found between our approach and DMRs identified using bisulphite conversion, methylationsensitive restriction digestion and microarrays (Old *et al.*, 2007; Chim *et al.*, 2008; Chu *et al.*, 2009; Bunce *et al.*, 2012; Yin *et al.*, 2012; Lun *et al.*, 2013; Hatt *et al.*, 2015). More concordant results were observed between our study and DMRs identified using MeDIP approaches (Papageorgiou *et al.*, 2009; Ioannides *et al.*, 2014; Xiang *et al.*, 2014).

## 4. Discussion

In this study, we undertook the genomewide biomarker discovery of DMRs between fetal and maternal DNA and confirmed the presence of a subset of these DMRs by combining for the first time MeDIP with insolution targeted enrichment and NGS.

Also, this is, to our knowledge, the first reported application of MeDIP in plasma samples. Previous studies have employed MeDIP in order to characterize the methylation status of different tissues using large amounts of input DNA. We were able to overcome this limitation and successfully enrich and characterize the methylome of multiple plasma samples using modifications of an existing MeDIP protocol (Borgel *et al.*, 2012). As a result, we developed a MeDIPNGS methodology that allowed us to use input concentrations of as low as 18–30 ng of plasma DNA derived from two 8-mL peripheral blood aliquots. This development constitutes another milestone in the development of an affordable epigenetic NIPT assay for the detection of fetal abnormalities in maternal plasma.

Previous studies have employed different methods for the discovery of fetalspecific biomarkers using methylation differences between fetal and maternal DNA, including sodium bisulphite conversion, methylationsensitive restriction digestion or affinitybased techniques (Gitan *et al.*, 2002; Chim *et al.*, 2005; Old *et al.*, 2007; Chim *et al.*, 2008; Laird, 2010; Tong *et al.*, 2010). Hypermethylated fetalspecific DMRs have been the focus of different studies due to their potential to be highly enriched and readily distinguished from the maternal background, and thus to be utilized for the detection of fetal aneuploidies (Chu *et al.*, 2009; Papageorgiou *et al.*, 2011; Tsaliki *et al.*, 2012; Yin *et al.*, 2012; Xiang *et al.*, 2014). In this study, we first undertook fetalspecific biomarker discovery between CVS, WBF and PL using MeDIPNGS (Fig. 1(*a*)). Subsequently, 331 DMRs (Table 4 & Table S1) were selected and subjected to MeDIP followed by targeted enrichment of eight CVS, eight WBF and eight PL samples (Fig. 1(*b*)). Pairwise comparisons on the three tissues based on their methylation levels confirmed that the overall methylation status of WBF and PL was significantly lower for the tested DMRs compared to the CVS samples. Further characterization of selected DMRs can therefore be performed on CVS and female plasma samples, since cffDNA is present in higher amounts in maternal plasma than in peripheral blood (Lo *et al.*, 1998).

Comparison of the selected DMRs with previous reports showed that the methylation patterns of several DMRs that were confirmed in our study are consistent with other methylationbased approaches (Old *et al.*, 2007; Chim *et al.*, 2008; Chu *et al.*, 2009; Bunce *et al.*, 2012; Yin *et al.*, 2012; Lun *et al.*, 2013; Hatt *et al.*, 2015), demonstrating that our novel, first reported, doubleenrichment approach is a robust method for the efficient and comprehensive characterization of selected DMRs. Further validation experiments are necessary in order to investigate the methylation variability in a larger cohort. It is also notable that our results show higher concordance with the results of other MeDIPbased approaches, reaffirming the reproducible nature of the MeDIP methodology (Papageorgiou *et al.*, 2009; Tsaliki *et al.*, 2012; Ioannides *et al.*, 2014; Xiang *et al.*, 2014).

Based on the characteristics of the validated DMRs and due to the great potential of this approach to be utilized in the clinical setting for the detection of the most common aneuploidies, future work will focus on the identification and characterization of additional DMRs on chromosomes 13, 18 and 21. In addition, the discovery of DMRs across all autosomes using our approach opens the way for identifying and validating markers associated with subchromosomal copy number changes, such us clinically relevant microdeletions and microduplications.

## Supplementary material

For supplementary material accompanying this paper visit https://doi.org/10.1017/S0016672316000136. click here to view supplementary material
