# Supplementary material for: Whole-genome fetal and maternal DNA methylation analysis using MeDIP-NGS for the identification of differentially methylated regions
Source: Genet Res (Camb). 2016 Nov 11;98:e15. doi: 10.1017/S0016672316000136 (PMC6865150; doi:10.1017/S0016672316000136)
Supplement: Supplementary file 1 [file S0016672316000136sup001.docx]

*Genomics Research*

Whole Genome Fetal and Maternal DNA Methylation Analysis using MeDIP-NGS for the Identification of Differentially Methylated Regions

Anna Keravnou, Marios Ioannides, Kyriakos Tsangaras, Charalambos Loizides, Michael D. Hadjidaniel, Elisavet A Papageorgiou, Skevi Kyriakou, Pavlos Antoniou, Petros Mina, Achilleas Achilleos, Maria Neofytou, Elena Kypri, Carolina Sismani, George Koumbaris, Philippos C Patsalis

Table S1. Chromosomal location of 331 selected fetal specific DMRs

| **DMR** | **Chromosome** | **Location** |
| --- | --- | --- |
| 1 | chr1 | 1p36.31 |
| 2 | chr1 | 1p36.23 |
| 3 | chr1 | 1p36.22 |
| 4 | chr1 | 1p36.13 |
| 5 | chr1 | 1p36.13 |
| 6 | chr1 | 1p36.13 |
| 7 | chr1 | 1p36.11 |
| 8 | chr1 | 1p34.3 |
| 9 | chr1 | 1p34.3 |
| 10 | chr1 | 1p34.2 |
| 11 | chr1 | 1p34.1 |
| 12 | chr1 | 1p34.1 |
| 13 | chr1 | 1p33 |
| 14 | chr1 | 1p32.3 |
| 15 | chr1 | 1p32.3 |
| 16 | chr1 | 1p32.1 |
| 17 | chr1 | 1p22.2 |
| 18 | chr1 | 1p22.2 |
| 19 | chr1 | 1p22.2 |
| 20 | chr1 | 1p21.3 |
| 21 | chr1 | 1p21.2 |
| 22 | chr1 | 1p12 |
| 23 | chr1 | 1q21.2 |
| 24 | chr1 | 1q22 |
| 25 | chr1 | 1q23.1 |
| 26 | chr1 | 1q24.1 |
| 27 | chr1 | 1q24.2 |
| 28 | chr1 | 1q25.3 |
| 29 | chr1 | 1q31.3 |
| 30 | chr1 | 1q41 |
| 31 | chr1 | 1q41 |
| 32 | chr1 | 1q41 |
| 33 | chr1 | 1q42.3 |
| 34 | chr1 | 1q44 |
| 35 | chr2 | 2p21 |
| 36 | chr2 | 2p14 |
| 37 | chr2 | 2p14 |
| 38 | chr2 | 2p14 |
| 39 | chr2 | 2p13.3 |
| 40 | chr2 | 2q24.2 |
| 41 | chr2 | 2q31.1 |
| 42 | chr2 | 2q32.3 |
| 43 | chr2 | 2q36.1 |
| 44 | chr2 | 2q37.1 |
| 45 | chr3 | 3p25.1 |
| 46 | chr3 | 3p22.1 |
| 47 | chr3 | 3p14.3 |
| 48 | chr3 | 3p14.3 |
| 49 | chr3 | 3p14.2 |
| 50 | chr3 | 3p14.1 |
| 51 | chr3 | 3q26.32 |
| 52 | chr3 | 3q27.1 |
| 53 | chr3 | 3q28 |
| 54 | chr3 | 3q29 |
| 55 | chr4 | 4p15.33 |
| 56 | chr4 | 4p15.1 |
| 57 | chr4 | 4p14 |
| 58 | chr4 | 4p13 |
| 59 | chr4 | 4q21.23 |
| 60 | chr4 | 4q21.23 |
| 61 | chr4 | 4q26 |
| 62 | chr4 | 4q28.1 |
| 63 | chr4 | 4q31.1 |
| 64 | chr4 | 4q31.3 |
| 65 | chr4 | 4q32.1 |
| 66 | chr4 | 4q35.1 |
| 67 | chr5 | 5p13.1 |
| 68 | chr5 | 5q11.1 |
| 69 | chr5 | 5q14.1 |
| 70 | chr5 | 5q14.1 |
| 71 | chr5 | 5q15 |
| 72 | chr5 | 5q23.2 |
| 73 | chr5 | 5q31.1 |
| 74 | chr5 | 5q31.1 |
| 75 | chr5 | 5q31.3 |
| 76 | chr5 | 5q33.2 |
| 77 | chr5 | 5q34 |
| 78 | chr5 | 5q35.1 |
| 79 | chr6 | 6p24.3 |
| 80 | chr6 | 6p24.1 |
| 81 | chr6 | 6p22.3 |
| 82 | chr6 | 6p22.3 |
| 83 | chr6 | 6p12.3 |
| 84 | chr6 | 6p12.3 |
| 85 | chr6 | 6p12.3 |
| 86 | chr6 | 6q16.3 |
| 87 | chr6 | 6q21 |
| 88 | chr6 | 6q21 |
| 89 | chr6 | 6q21 |
| 90 | chr6 | 6q23.3 |
| 91 | chr6 | 6q25.3 |
| 92 | chr7 | 7p21.1 |
| 93 | chr7 | 7p21.1 |
| 94 | chr7 | 7p15.3 |
| 95 | chr7 | 7p15.1 |
| 96 | chr7 | 7p14.3 |
| 97 | chr7 | 7p14.3 |
| 98 | chr7 | 7p14.2 |
| 99 | chr7 | 7p14.2 |
| 100 | chr7 | 7p14.2 |
| 101 | chr7 | 7p14.2 |
| 102 | chr7 | 7p14.1 |
| 103 | chr7 | 7p14.1 |
| 104 | chr7 | 7p14.1 |
| 105 | chr7 | 7p14.1 |
| 106 | chr7 | 7p14.1 |
| 107 | chr7 | 7p14.1 |
| 108 | chr7 | 7p13 |
| 109 | chr7 | 7p12.3 |
| 110 | chr7 | 7p12.1 |
| 111 | chr7 | 7q11.23 |
| 112 | chr7 | 7q11.23 |
| 113 | chr7 | 7q21.3 |
| 114 | chr7 | 7q22.1 |
| 115 | chr7 | 7q22.1 |
| 116 | chr7 | 7q31.31 |
| 117 | chr7 | 7q31.32 |
| 118 | chr7 | 7q36.1 |
| 119 | chr7 | 7q36.2 |
| 120 | chr8 | 8p21.3 |
| 121 | chr8 | 8p21.2 |
| 122 | chr8 | 8p21.2 |
| 123 | chr8 | 8p21.2 |
| 124 | chr8 | 8p21.1 |
| 125 | chr8 | 8q12.1 |
| 126 | chr8 | 8q22.2 |
| 127 | chr8 | 8q22.2 |
| 128 | chr8 | 8q23.3 |
| 129 | chr8 | 8q23.3 |
| 130 | chr9 | 9p13.2 |
| 131 | chr9 | 9q22.31 |
| 132 | chr9 | 9q22.32 |
| 133 | chr9 | 9q22.32 |
| 134 | chr9 | 9q31.3 |
| 135 | chr9 | 9q33.3 |
| 136 | chr9 | 9q33.3 |
| 137 | chr9 | 9q34.11 |
| 138 | chr9 | 9q34.13 |
| 139 | chr10 | 10p15.1 |
| 140 | chr10 | 10p14 |
| 141 | chr10 | 10p14 |
| 142 | chr10 | 10p14 |
| 143 | chr10 | 10p14 |
| 144 | chr10 | 10p14 |
| 145 | chr10 | 10p14 |
| 146 | chr10 | 10p13 |
| 147 | chr10 | 10p13 |
| 148 | chr10 | 10p11.22 |
| 149 | chr10 | 10q21.3 |
| 150 | chr10 | 10q24.1 |
| 151 | chr10 | 10q24.2 |
| 152 | chr10 | 10q25.2 |
| 153 | chr10 | 10q25.3 |
| 154 | chr11 | 11p15.4 |
| 155 | chr11 | 11q13.3 |
| 156 | chr11 | 11q14.1 |
| 157 | chr11 | 11q14.2 |
| 158 | chr11 | 11q14.2 |
| 159 | chr11 | 11q21 |
| 160 | chr11 | 11q23.3 |
| 161 | chr11 | 11q24.2 |
| 162 | chr11 | 11q24.3 |
| 163 | chr11 | 11q25 |
| 164 | chr12 | 12p13.32 |
| 165 | chr12 | 12p13.31 |
| 166 | chr12 | 12p13.2 |
| 167 | chr12 | 12p13.2 |
| 168 | chr12 | 12p12.3 |
| 169 | chr12 | 12p12.1 |
| 170 | chr12 | 12q13.12 |
| 171 | chr12 | 12q13.12 |
| 172 | chr12 | 12q13.13 |
| 173 | chr12 | 12q24.21 |
| 174 | chr12 | 12q24.21 |
| 175 | chr12 | 12q24.31 |
| 176 | chr13 | 13q12.11 |
| 177 | chr13 | 13q12.2 |
| 178 | chr13 | 13q12.2 |
| 179 | chr13 | 13q12.3 |
| 180 | chr13 | 13q13.1 |
| 181 | chr13 | 13q13.1 |
| 182 | chr13 | 13q14.11 |
| 183 | chr13 | 13q14.11 |
| 184 | chr13 | 13q14.2 |
| 185 | chr13 | 13q14.2 |
| 186 | chr13 | 13q14.2 |
| 187 | chr13 | 13q14.3 |
| 188 | chr13 | 13q14.3 |
| 189 | chr13 | 13q14.3 |
| 190 | chr13 | 13q21.33 |
| 191 | chr13 | 13q31.2 |
| 192 | chr13 | 13q32.1 |
| 193 | chr13 | 13q32.1 |
| 194 | chr13 | 13q32.3 |
| 195 | chr13 | 13q32.3 |
| 196 | chr13 | 13q32.3 |
| 197 | chr13 | 13q33.1 |
| 198 | chr13 | 13q34 |
| 199 | chr13 | 13q34 |
| 200 | chr13 | 13q34 |
| 201 | chr13 | 13q34 |
| 202 | chr13 | 13q34 |
| 203 | chr14 | 14q13.3 |
| 204 | chr14 | 14q21.1 |
| 205 | chr14 | 14q21.1 |
| 206 | chr14 | 14q22.2 |
| 207 | chr14 | 14q23.1 |
| 208 | chr14 | 14q24.3 |
| 209 | chr14 | 14q24.3 |
| 210 | chr14 | 14q31.3 |
| 211 | chr14 | 14q32.11 |
| 212 | chr14 | 14q32.12 |
| 213 | chr14 | 14q32.12 |
| 214 | chr15 | 15q14 |
| 215 | chr15 | 15q22.31 |
| 216 | chr15 | 15q22.33 |
| 217 | chr15 | 15q22.33 |
| 218 | chr15 | 15q23 |
| 219 | chr15 | 15q23 |
| 220 | chr15 | 15q24.1 |
| 221 | chr15 | 15q26.1 |
| 222 | chr15 | 15q26.1 |
| 223 | chr16 | 16p13.13 |
| 224 | chr16 | 16p13.13 |
| 225 | chr16 | 16p12.1 |
| 226 | chr16 | 16p11.2 |
| 227 | chr16 | 16q12.1 |
| 228 | chr16 | 16q12.1 |
| 229 | chr16 | 16q12.2 |
| 230 | chr17 | 17p13.3 |
| 231 | chr17 | 17p13.1 |
| 232 | chr17 | 17p11.2 |
| 233 | chr17 | 17q11.2 |
| 234 | chr17 | 17q12 |
| 235 | chr17 | 17q23.3 |
| 236 | chr17 | 17q24.2 |
| 237 | chr17 | 17q24.2 |
| 238 | chr17 | 17q24.2 |
| 239 | chr17 | 17q24.3 |
| 240 | chr18 | 18p11.31 |
| 241 | chr18 | 18p11.31 |
| 242 | chr18 | 18p11.31 |
| 243 | chr18 | 18p11.31 |
| 244 | chr18 | 18p11.22 |
| 245 | chr18 | 18p11.22 |
| 246 | chr18 | 18p11.22 |
| 247 | chr18 | 18p11.22 |
| 248 | chr18 | 18q11.2 |
| 249 | chr18 | 18q11.2 |
| 250 | chr18 | 18q11.2 |
| 251 | chr18 | 18q12.1 |
| 252 | chr18 | 18q12.1 |
| 253 | chr18 | 18q12.1 |
| 254 | chr18 | 18q12.2 |
| 255 | chr18 | 18q12.3 |
| 256 | chr18 | 18q21.1 |
| 257 | chr18 | 18q21.1 |
| 258 | chr18 | 18q21.1 |
| 259 | chr18 | 18q21.2 |
| 260 | chr18 | 18q21.2 |
| 261 | chr18 | 18q21.2 |
| 262 | chr18 | 18q21.31 |
| 263 | chr18 | 18q21.31 |
| 264 | chr18 | 18q21.32 |
| 265 | chr18 | 18q21.32 |
| 266 | chr18 | 18q21.32 |
| 267 | chr18 | 18q21.33 |
| 268 | chr18 | 18q21.33 |
| 269 | chr18 | 18q21.33 |
| 270 | chr18 | 18q22.3 |
| 271 | chr19 | 18p13.12 |
| 272 | chr19 | 19q12 |
| 273 | chr19 | 19q13.12 |
| 274 | chr19 | 19q13.13 |
| 275 | chr19 | 19q13.13 |
| 276 | chr19 | 19q13.2 |
| 277 | chr20 | 20p11.23 |
| 278 | chr20 | 20p11.23 |
| 279 | chr20 | 20p11.22 |
| 280 | chr20 | 20p11.22 |
| 281 | chr20 | 20q11.23 |
| 282 | chr20 | 20q13.2 |
| 283 | chr20 | 20q13.31 |
| 284 | chr20 | 20q13.31 |
| 285 | chr20 | 20q13.32 |
| 286 | chr21 | 21q11.2 |
| 287 | chr21 | 21q21.1 |
| 288 | chr21 | 21q21.1 |
| 289 | chr21 | 21q21.1 |
| 290 | chr21 | 21q21.1 |
| 291 | chr21 | 21q21.3 |
| 292 | chr21 | 21q21.3 |
| 293 | chr21 | 21q21.3 |
| 294 | chr21 | 21q21.3 |
| 295 | chr21 | 21q21.3 |
| 296 | chr21 | 21q21.3 |
| 297 | chr21 | 21q21.3 |
| 298 | chr21 | 21q22.11 |
| 299 | chr21 | 21q22.11 |
| 300 | chr21 | 21q22.11 |
| 301 | chr21 | 21q22.11 |
| 302 | chr21 | 21q22.12 |
| 303 | chr21 | 21q22.13 |
| 304 | chr21 | 21q22.13 |
| 305 | chr21 | 21q22.13 |
| 306 | chr21 | 21q22.13 |
| 307 | chr21 | 21q22.2 |
| 308 | chr21 | 21q22.2 |
| 309 | chr21 | 21q22.2 |
| 310 | chr21 | 21q22.2 |
| 311 | chr21 | 21q22.2 |
| 312 | chr21 | 21q22.2 |
| 313 | chr21 | 21q22.2 |
| 314 | chr21 | 21q22.2 |
| 315 | chr21 | 21q22.3 |
| 316 | chr21 | 21q22.3 |
| 317 | chr21 | 21q22.3 |
| 318 | chr21 | 21q22.3 |
| 319 | chr21 | 21q22.3 |
| 320 | chr21 | 21q22.3 |
| 321 | chr21 | 21q22.3 |
| 322 | chr21 | 21q22.3 |
| 323 | chr21 | 21q22.3 |
| 324 | chr21 | 21q22.3 |
| 325 | chr21 | 21q22.3 |
| 326 | chr22 | 22q12.2 |
| 327 | chr22 | 22q13.1 |
| 328 | chr22 | 22q13.1 |
| 329 | chr22 | 22q13.2 |
| 330 | chr22 | 22q13.2 |
| 331 | chr22 | 22q13.2 |
